# Identification and Functional Analysis of *tgfb2b* Gene in Ovarian Development of Chinese Tongue Sole (*Cynoglossus semilaevis*)

**DOI:** 10.3390/biom16010105

**Published:** 2026-01-07

**Authors:** Xihong Li, Kaili Zhang, Yue Zhang, Zhijie Li, Zhangfan Chen, Hongyan Wang, Songlin Chen, Na Wang

**Affiliations:** 1 State Key Laboratory of Mariculture Biobreeding and Sustainable Goods, Yellow Sea Fisheries Research Institute, Chinese Academy of Fishery Sciences, Qingdao 266071, China; 2 Laboratory for Marine Fisheries Science and Food Production Processes, Qingdao Marine Science and Technology Center, Qingdao 266237, China

**Keywords:** *Cynoglossus semilaevis*, *tgfb2b*, ovarian development, siRNA, miRNA, *foxl2-cyp19a-esr*

## Abstract

Transforming growth factor β (TGF-β) superfamily members are critical in teleost sex determination and differentiation. *Tgfb2b* is an important TGF-β ligand gene exhibiting dominant expression in the ovary of Chinese tongue sole (*Cynoglossus semilaevis*), yet its function in sex regulation remains unclear. In the present study, the gene expression pattern, transcriptional regulation, and knockdown effect were examined. Its expression persisted and showed a gradual increase throughout ovarian development from 3 months to 1.5 years post-hatching. *In situ* hybridization (ISH) revealed that the gene was distributed across oocytes at stages I–III, while scarcely detectable in the testis. The transcriptional factors CCAAT/enhancer binding protein α (C/EBPα) and Jun proto-oncogene AP-1 transcription factor subunit (c-Jun) could repress the activity of *tgfb2b* promoter. *In vitro* knockdown of *tgfb2b* in *C. semilaevis* ovarian cells led to downregulation of its downstream genes (e.g., *smad1* and *smad2*) as well as other sex-related genes (e.g., *foxl2* and *esr2b*). Moreover, multi-omics analysis indicated that, in *C. semilaevis* gonads, a miRNA named novel-m0083-3p showed an opposite expression pattern with *tgfb2b* and might have a binding site with the gene. By dual-luciferase assay, *tgfb2b* was validated to be directly targeted and suppressed by the miRNA. These results demonstrate that *tgfb2b* plays a significant role in ovarian differentiation and development. Further functional and molecular studies on the interplay between *tgfb2b* and the *foxl2–cyp19a–esr* axis will help elucidate the regulatory network underlying sex development in teleost.

## 1. Introduction

As an economically important mariculture fish, the Chinese tongue sole (*Cynoglossus semilaevis*) has a female heterogametic ZW sex-determining system [1,2]. It exhibits sexual dimorphism that the females mature later and grow 2–4 times bigger than the males [2]. However, a high percentage of the males in farmed populations give rise to huge production and economic losses. Explaining the molecular mechanism underlying female sex formation and making effort for high-female-ratio fry will be helpful for the sustainable development of the aquaculture.

It is well known that sex development (including sex determination, differentiation, and maturation) is an important process, by which undifferentiated gonad becomes a testis or an ovary. It can be controlled genetically (genetic sex determination, GSD), environmentally (environmental sex determination, ESD), or by a combination of the two categories [3]. In fish species with GSD, various sex-determining genes have been identified. Several of them (e.g., *dmy*, *dmrt1*, and *sox3-Y*) are transcription factors, while many others including *anti-Müllerian hormone* (*amh*) *receptor type II* (*amhr2*), Y chromosome-linked duplicate of *amh* (*amhy*), *gonadal soma-derived factor* (*gsdf*), *growth differentiation factor 6* (*gdf6*), and *bone morphogenetic protein receptor* (*bmpr*) are tightly associated with transforming growth factor β (TGF-β) superfamily [3,4,5]. Even in *Oryzias latipes* and *O. dancena* that separately utilize *dmy* and *sox3-Y* as sex-determining gene, mutations of the superfamily genes *gsdf* and *amhr2* could cause dysregulation of testis/ovary development [6,7]. Other members of the superfamily such as *gdf9*, *bmp15*, and *glial cell line-derived neurotrophic factor* (*gdnf*) are also widely researched to be instrumental in sex differentiation [8,9,10]. For example, the oocyte-secreted factors *gdf9* and *bmp15* play important roles in follicle development, oocyte maturation, and steroidogenesis [8,9]. In zebrafish, *gdf9* is presented in differentiating or differentiated gonads, with an expression pattern closely relating to ovarian development [11]. Knockdown of this gene decreases the number of female fish, indicating its involvement in facilitating oocyte/ovary differentiation [11]. *Bmp15* deficiency in females also leads to sex reversal in the mid- to late-juvenile stage and results in fertile males, which implies the requirement in female differentiation and maintenance [12]. Regarding the different isoforms of TGF-β ligand genes (*tgfb1*/*2*/*3*), *tgfb1* has been indicated to suppress zebrafish oocyte maturation, too [13]. However, those studies are mainly focused on part molecules of the superfamily, while relevant reports on *tgfb2* and *tgfb3* are still inadequate and need more investigations.

Due to genome duplication, *tgfb* genes often display paralog pairs (suffixing with “a” and “b”) in teleost [14]. Based on previous transcriptome data of *C. semilaevis* [15], we found five genes (*tgfb1a*, *tgfb2a*, *tgfb2b*, *tgfb3a*, and *tgfb3b*) and compared their expressions in somatotropic and reproductive tissues. Especially, *tgfb2b* was highest expressed in female gonad, with a significant sexually dimorphic pattern. In this study, to explore the potential role of *tgfb2b* in fish ovarian differentiation and development, the gene sequence was cloned and its expression profiles in different tissues and at different developmental periods were analyzed. *In situ* hybridization, promoter transcriptional activity, small interfering RNA (siRNA)-mediated knockdown effect, and microRNA (miRNA)-targeted regulation of the gene were also characterized. The results would contribute to better understanding the biological function of TGF-β superfamily in teleost.

## 2. Materials and Methods

### 2.1. Fish Samples

Fish individuals used for this study were cultured at Haiyang breeding base (Shandong, China). Before the experiments, they were anesthetized with MS-222 to reduce the pain. Genetic sex of the fish was determined using the previously reported method with the primers sex-F and sex-R (Table 1) [16]. Tissues including kidney, gonad, brain, muscle, liver, and spleen were randomly sampled from three female and three male 1-year-old (1 Y) *C. semilaevis*. At the same time, gonads of the fish at different developmental periods, such as 3 months, 7 months, 1 year, and 1.5 years post-hatching (3 M, 7 M, 1 Y, and 1.5 Y), were also gathered. Each period contained at least three female and three male individuals. The samples were subsequently stored at −80 °C until RNA isolation. The fish experiment was inspected and approved by the Animal Care and Use Committee at the Yellow Sea Fisheries Research Institute, Chinese Academy of Fishery Sciences (Approve No.: YSFRI-2023006, approval date: 2 March 2023).

### 2.2. Cell Culture

Human embryonal kidney (HEK) 293T cells (ATCC^®^ CRL-3216™) purchased from ATCC (Manassas, VA, USA) and *C. semilaevis* ovarian (CSO) cells established by our lab [17] were used in this study. HEK 293T cells were grown in DME/F-12 medium added with 10% fetal bovine serum (FBS) (Gibco, Auckland, New Zealand) at 37 °C in a 5% CO_2_ incubator. L-15 medium comprising 15% FBS and bFGF (Invitrogen, Carlsbad, CA, USA) was selected for CSO cells, and the cells were cultured at 24 °C. One day prior to transfection, the cells were seeded in 12- or 24-well plates and allowed to grow to a density of 70–80%.

### 2.3. RNA Isolation and cDNA Synthesis

Total RNA of the tissues or cells was isolated using TRIzol reagent (Invitrogen). RNA quantity and quality was assessed via a NanoDrop 2000 spectrophotometer (Thermo Fisher Scientific, Wilmington, DE, USA) and agarose gel electrophoresis. Reverse transcription from the purified RNA to complementary DNA (cDNA) was conducted using HiScript^®^ III RT SuperMix with gDNA wiper (Vazyme, Nanjing, China), following the product instruction.

### 2.4. Gene Sequence Cloning and Analysis

According to the sequence information of *tgfb2b* in GenBank (ID: 103390834), specific primers *tgfb2b*-CDS-F and *tgfb2b*-CDS-R (Table 1) were designed to clone its coding sequence (CDS). The amplification product was ligated into pEASY-T1 vector (TransGen, Beijing, China) and sequenced by Qingdao Branch of TsingKe Biotech Co., Ltd. (Qingdao, China). Predicted domain of the sequence was analyzed using NCBI Conserved Domain Database (CDD, https://www.ncbi.nlm.nih.gov/cdd (accessed on 9 November 2023)) and SMART (http://smart.emblheidelberg.de/ (accessed on 9 November 2023)). After BLAST search at NCBI (http://www.ncbi.nlm.nih.gov/BLAST/ (accessed on 9 November 2023)), homologous amino acid sequences of different TGF-β isoforms from seven fish species (*Danio rerio*, *Oryzias latipes*, *Oreochromis niloticus*, *Takifugu rubripes*, *Ctenopharyngodon idella*, *Ictalurus punctatus*, and *Anarrhichthys ocellatus*) and three common non-fish species (*Homo sapiens*, *Mus musculus*, and *Gallus gallus*) were acquired (Supplementary Table S1). Multiple sequence alignment was completed adopting ClustalW program. A phylogenetic tree was generated by MEGA7 software (version 7.0.26) using Neighbor-Joining method with 1000 bootstrap replicates. Then, the tree was beautified and visualized in EvolView (http://www.evolgenius.info/evolview (accessed on 9 November 2023)). In addition, the normalized fragments per kilo base of transcript per million mapped reads (FPKM) values of *C. semilaevis tgfb* genes were obtained from our previous transcriptome data [15]. According to the values, an expression heatmap was drawn on Omicshare website (https://www.omicshare.com/tools (accessed on 9 November 2023)).

### 2.5. Expression Detection in Different Tissues and Periods

The expression patterns of *tgfb2b* in different tissues and at different developmental periods of female and male *C. semilaevis* were detected through quantitative real-time (qPCR) analysis. The above synthesized cDNA from 6 tissues (kidney, gonad, brain, muscle, liver, and spleen) at 1 Y and from the gonads at 4 developmental periods (3 M, 7 M, 1 Y, and 1.5 Y) were used for the analysis. The reliable reference gene *β-actin* was selected as internal control, and the primers were listed in Table 1. According to the standardized procedures, the reactions were performed using THUNDERBIRD™ Next SYBR^®^ qPCR Mix (TOYOBO, Tokyo, Japan) on a Quant Gene 9600 instrument (BIOER, Hangzhou, China). Each experiment included at least three biological replicates. Gene relative expression level was assessed using 2^−ΔΔCt^ method [18]. The data was presented as mean ± standard deviation (mean ± SD). Statistical analysis was performed by one-way analysis of variance (ANOVA) and least significant difference (LSD) multiple comparison with the online OmicShare tool (https://www.omicshare.com/tools (accessed on 18 April 2024)). *p* < 0.05 was accepted for significant difference.

### 2.6. In Situ Hybridization (ISH)

Ovary and testis sections of 1 Y *C. semilaevis* were prepared by our lab. Briefly, parts of the gonads were fixed using 4% paraformaldehyde, dehydrated by graded ethanol, embedded in paraffin, and cut into slices of about 5 μm thickness. A fragment (609 bp in length) of *tgfb2b* CDS was cloned with a pair of probe primers *tgfb2b*-SP6-F and *tgfb2b*-T7-R (Table 1). The amplified fragment was used to produce digoxigenin (DIG)-labeled antisense/sense probe with T7/SP6 RNA polymerase (Roche, Mannheim, Germany). Afterwards, the RNA probes were purified by lithium chloride (LiCl) precipitation. The detailed operation steps of ISH were elaborated in our previous studies [8]. Finally, the signals were examined by BCIP/NBT Chromogen Kit (Solarbio, Beijing, China) and photographed on a Nikon Eclipse 80i microscope (Nikon, Tokyo, Japan).

### 2.7. Promoter Amplification and Activity Analysis

The predicted promoter sequence (2041 bp upstream) of *tgfb2b* was amplified with the infusion primers *tgfb2b*-pF and *tgfb2b*-pR (Table 1). Using the One Step Fusion Cloning Mix (Toroivd, Shanghai, China), the amplification product was ligated into HindIII-digested pGL3-basic vector (Promega, Madison, WI, USA) to generate a recombinant luciferase reporter plasmid called pGL3-*tgfb2b*-pro. Then, the plasmid was transfected into HEK 293T cells in a 24-well plate. pGL3-basic (negative control) and pGL3-control (positive control) were also transfected to the cells, respectively. Each group contained 4 wells, and pRL-TK was used as internal reference. Lipo8000™ Transfection Reagent (Beyotime, Shanghai, China) was selected for the transfection referring to the instruction. After transfection for approximately 48 h, cells were harvested to asses firefly and Renilla luciferase activities by the Dual-Luciferase Reporter Gene Assay Kit (Beyotime) and a microplate reader (Thermo, Vantaa, Finland). The relative luciferase activity of each group was calculated as the average ratio of firefly to Renilla luciferase activity. Statistical analysis was performed the same as described in Section 2.5.

At the same time, transcription factor (TF) binding sites in *tgfb2b* promoter were searched on PROMO website (http://alggen.lsi.upc.es/ (accessed on 20 November 2023)). CDSs of possible TFs were amplified from *C. semilaevis* and inserted into pcDNA3.1 vector. In addition, point mutations of the binding sites in *tgfb2b* promoter were realized by Fast Site-Directed Mutagenesis Kit (Tiangen, Beijing, China). The primers were listed in Table 1. Cell co-transfections with pGL3-*tgfb2b*-pro (wild- or mutant-type) and TF plasmids were conducted in 24-well plates, following the procedure of Lipo8000™ Transfection Reagent (Beyotime). Each group contained 4 wells, too. Luciferase assay and statistical analysis were carried out with the above-mentioned methods.

### 2.8. RNA Interference (RNAi)

Three siRNA oligonucleotides (Table 2) targeting *tgfb2b* gene were designed and synthesized from Sangon Biotech Co., Ltd. (Shanghai, China). They were separately transfected into CSO cells with the help of riboFECT^TM^ CP Transfection Kit (Ribobio, Guangzhou, China). A randomly synthesized and nonspecific siRNA from the same company was applied as the negative control (NC). In addition, cyanin 3 (cy3)-labeled siRNA was used to determine the transfection efficiency by observing cy3 fluorescence under a fluorescence microscope (Olympus, Tokyo, Japan). The cells were collected after transfection for 24 h. RNA extraction, cDNA synthesis, and qPCR assay were subsequently carried out with the same methods as described above. Expression levels of *tgfb2b* and other related genes such as SMAD family members (*smad1* and *smad2*), *forkhead box L2* (*foxl2*), and *estrogen receptor 2b* (*esr2b*) were assessed using 2^−ΔΔCt^ method [18]. Statistical analysis between siRNA-transfected group and NC group was performed by *t*-test. *p* < 0.05 or *p* < 0.01 was considered as significant difference or extremely significant difference, respectively.

### 2.9. miRNA Prediction, Validation, and Transfection

Our whole transcriptomic data analysis [19] suggested that *tgfb2b* might be regulated by the miRNA novel-m0083-3p. Expression bar graphs of the miRNA and *tgfb2b* in *C. semilaevis* gonads were plotted based on the transcriptomic data [19]. To further verify the relationship between *tgfb2b* and the miRNA, a 3′-untranslated (3′-UTR) fragment of *tgfb2b* that comprised the binding site with novel-m0083-3p was cloned using the primers *tgfb2b*-0083-F *and tgfb2b*-0083-R (Table 1). The sequence was double-digested with XhoI and SalI and ligated into pmir-GLO vector, generating a GLO-*tgfb2b*-WT reporter plasmid. A mutant plasmid called GLO-*tgfb2b*-MT was also obtained by use of Fast Site-Directed Mutagenesis Kit (Tiangen) and the mutation primers (*tgfb2b*-0083-muF and *tgfb2b*-0083-muR, Table 1). Meanwhile, novel-m0083-3p mimics (Table 2) and a random-synthesized negative control (NC) were ordered from Sangon Biotech (Shanghai). Using Lipo8000™ Transfection Reagent (Beyotime), GLO-*tgfb2b*-WT/MT and novel-m0083-3p mimics/NC were transfected to HEK 293T cells, respectively. Two days later, the luciferase activity was measured by Dual-Luciferase Reporter Gene Assay Kit (Beyotime). The experiment was conducted in three replicates. Statistical analysis between every two groups was performed the same as described in Section 2.8.

## 3. Results

### 3.1. Sequence Characterization of tgfb2b

**Figure 1:**
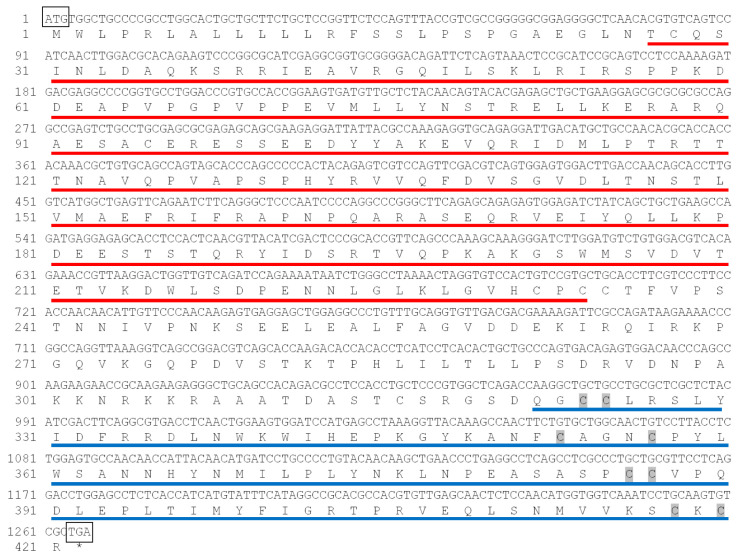
The CDS and deduced amino acid sequences of *tgfb2b*. The conserved cysteine residues were indicated in gray shadow; TGF-β propeptide region and TGF-β domain are indicated in red and blue lines, respectively. The asterisk represents the stop codon.

**Figure 2:**
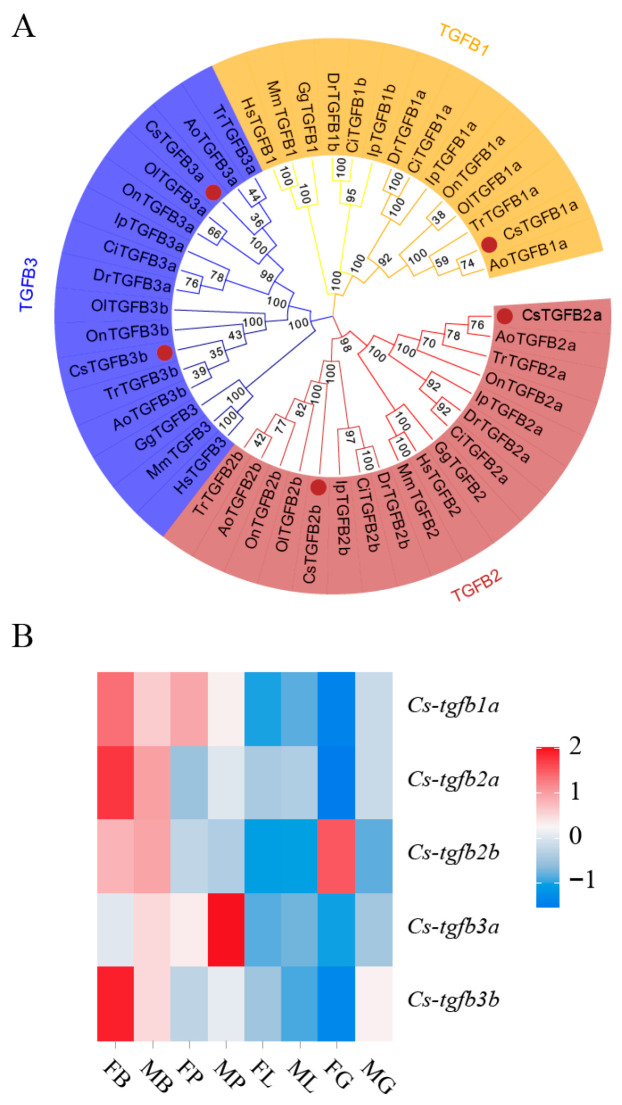
Phylogenetic tree and expression heatmap of *tgfb2b*. (**A**) Phylogenetic tree of TGF-β amino acid sequences from *C. semilaevis* (Cs), *Danio rerio* (Dr), *Oryzias latipes* (Ol), *Oreochromis niloticus* (On), *Takifugu rubripes* (Tr), *Ctenopharyngodon idella* (Ci), *Ictalurus punctatus* (Ip), *Anarrhichthys ocellatus* (Ao), *Gallus gallus* (Gg), *Mus musculus* (Mm) and *Homo sapiens* (Hs). The number on each branch indicates the bootstrap value using Neighbor-Joining method. (**B**) Expression heatmap of *tgfb* mRNA abundances in four tissues of female and male *C. semilaevis*. FB: female brain; MB: male brain; FP: female pituitary; MP: male pituitary; FG: female gonad; MG: male gonad; FL: female liver; ML: male liver. The expression levels are quantified as the normalized fragments per kilo base of transcript per million mapped reads (FPKM) of transcriptome data. Colors from red to blue indicate high to low expression.

As shown in Figure 1, CDS of *C. semilaevis tgfb2b* gene contained 1266 bp. It encoded 421 amino acids with a TGF-β propeptide region in 27–234 residues, a TGF-β domain in 322–420 residues, and 8 conserved cysteines (C). As expected, the phylogenetic tree (Figure 2A) displayed that various TGF-β amino acid sequences were clustered into three clades (TGFB1, TGFB2, and TGFB3 clades). Each clade can be further divided into two small groups (e.g., TGFB2a and TGFB2b). Among them, *C. semilaevis* TGFB2b was closely grouped with the homologous sequences from *O. latipes* and *I. punctatus*. Expression heatmap revealed that, among different isoforms of *C. semilaevis*, only *tgfb2b* gene was highly expressed in female gonad (Figure 2B).

### 3.2. Expression Pattern and Cyto-Location of tgfb2b

**Figure 3:**
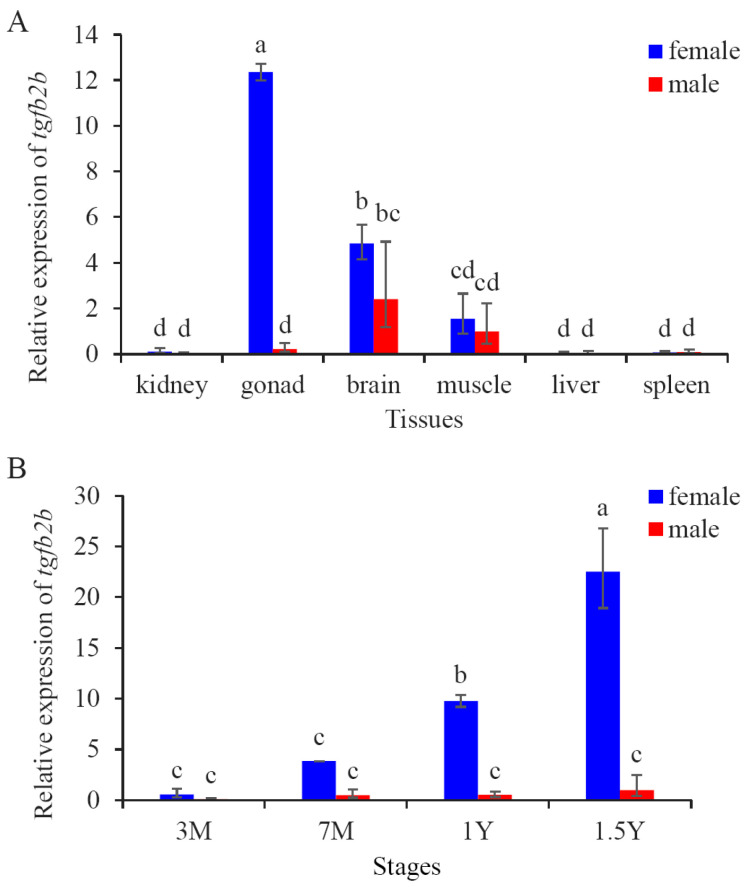
Expression profiles of *C. semilaevis tgfb2b* in different tissues and at different developmental periods. (**A**) Expression profile of *tgfb2b* in different tissues of 1-year-old (1 Y) *C. semilaevis*. (**B**) Expression profile of *tgfb2b* in gonads of *C. semilaevis* at different developmental periods. 3 M: 3-month-old; 7 M: 7-month-old; 1 Y: 1-year-old; 1.5 Y: 1.5-year-old. The data are presented as mean ± SD from three separate individuals (n = 3). Different letters above the bars indicate significant difference (*p* < 0.05).

To further evaluate the tissue expression of *tgfb2b*, qPCR experiments were completed in six tissues from 1 Y female and male *C. semilaevis*. The prominent expression of this gene appeared in ovary, but little expression was detected in testis (Figure 3A). It also showed moderate expression levels in brain and muscle, slightly higher in female than in male (Figure 3A).

The expression patterns of *tgfb2b* in gonads across distinct developmental periods were inspected, too. It was continuously expressed and showed gradual upregulation in ovaries from 3 M to 1.5 Y (Figure 3B). Nevertheless, the expression levels were constantly low in male gonads.

**Figure 4:**
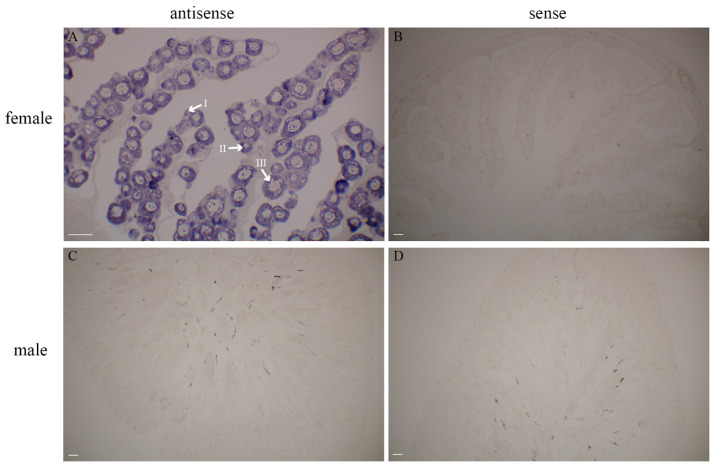
*In situ* localization of *tgfb2b* in 1 Y *C. semilaevis* gonads. (**A**) Female gonad with antisense probe for *tgfb2b*. Oocytes at different stages are indicated with I, II, and III. (**B**) Female gonad with sense probe for *tgfb2b*. (**C**) Male gonad with antisense probe for *tgfb2b*. (**D**) Male gonad with sense probe for *tgfb2b*. Scale bar = 100 μm.

ISH results displayed the cellular localization of *tgfb2b* in gonads. In the ovary of 1 Y female individual, oocytes at various stages (stage I, II, and III) were obviously seen (Figure 4A). By contrast, the gene was scarcely distributed in *C. semilaevis* testis (Figure 4C). No expression signals were discovered with sense probes (Figure 4B,D).

### 3.3. Transcriptional Regulation of tgfb2b Promoter Activity

**Figure 5:**
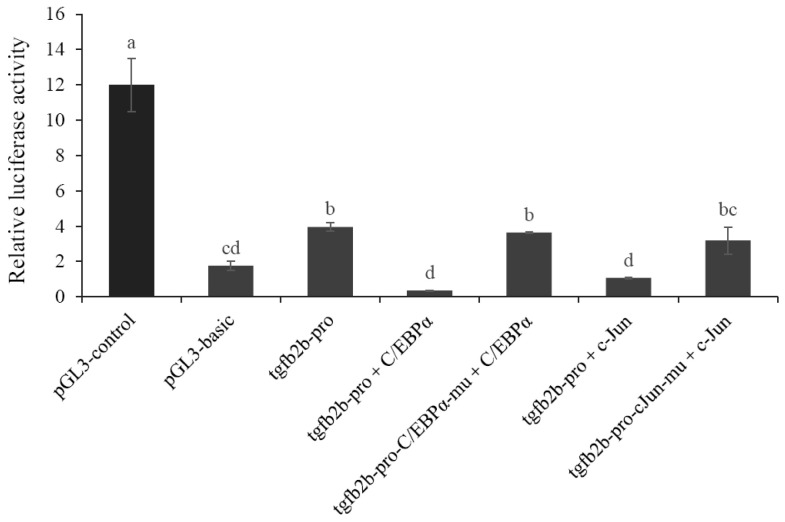
Transcriptional activity of *tgfb2b* promoter. Tgfb2b-pro: *C. semilaevis tgfb2b* promoter; mu: mutant. The data are presented as mean ± SD from four separate cell wells (n = 4). Different letters above the bars indicate significant difference (*p* < 0.05).

Dual-luciferase detection results (Figure 5) showed that relative luciferase activity of the cells transfected with *tgfb2b* promoter was 1.24-fold higher than that of pGL3-basic group, which indicated that it had a remarkable transcriptional activity in initiating gene expression. Moreover, the results also displayed that the activity could be significantly repressed by the TFs including CCAAT/enhancer binding protein α (C/EBPα) and Jun proto-oncogene AP-1 transcription factor subunit (c-Jun) (Figure 5). When the promoter sequence was mutated at C/EBPα or c-Jun binding site, the inhibitory effect was removed and the activity recovered back to the original level (Figure 5).

### 3.4. Knockdown Effect of tgfb2b siRNA in CSO Cells

**Figure 6:**
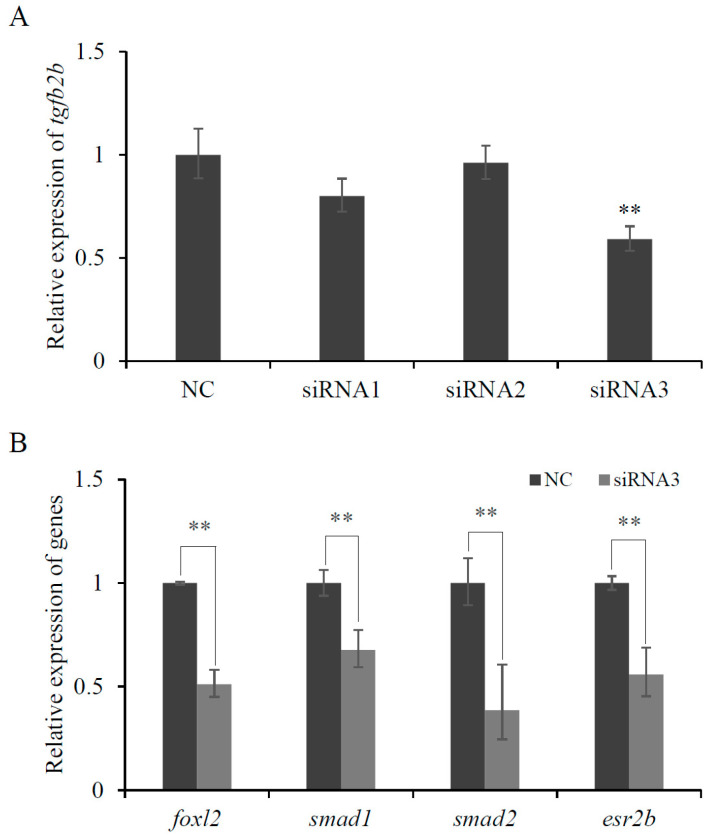
In vivo knockdown effect of *tgfb2b* siRNA. (**A**) Interference efficiency of *tgfb2b* siRNA. (**B**) Relative expression levels of genes after transfection of *tgfb2b* siRNA. The data are presented as mean ± SD (n = 3). NC, negative control group. Asterisks demonstrate significant differences (**: *p* < 0.01).

To explore the function of *tgfb2b*, three specific siRNAs were transfected into CSO cells. In siRNA3-transfected group, the expression of *tgfb2b* declined to about 59% of the control group (Figure 6A). It showed extremely significant knockdown effect (*p* < 0.01) (Figure 6A). Thereafter, the expressions of several related genes such as *foxl2*, *smad1*, *smad2*, and *esr2b* were also detected to be noticeably downregulated (Figure 6B).

### 3.5. The Influence of Novel-m0083-3p on tgfb2b

**Figure 7:**
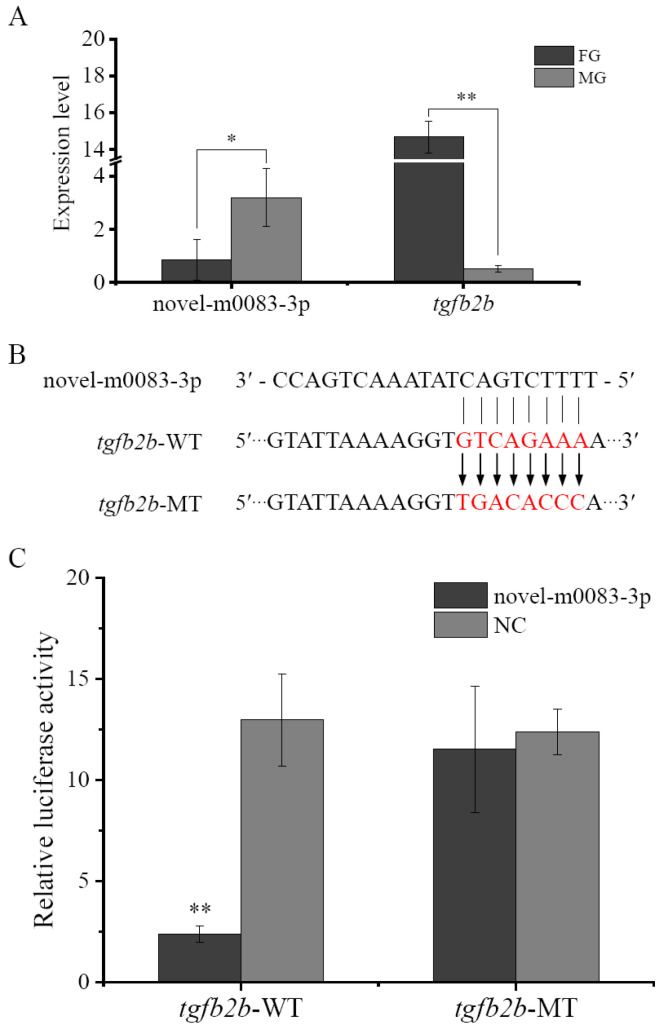
*Tgfb2b* is negatively regulated by novel-m0083-3p. (**A**) Expression levels of novel-m0083-3p and *tgfb2b* in gonads of 1.5Y *C. semilaevis*. (**B**) Putative binding site of novel-m0083-3p and *tgfb2b*. (**C**) Luciferase activities of HEK 293T cells co-transfected with *tgfb2b* recombinant plasmids and novel-m0083-3p mimics. WT: wild-type; MT: mutant-type. The data are presented as mean ± SD (n = 3). Asterisks demonstrate significant differences (*: *p* < 0.05; **: *p* < 0.01).

As shown in Figure 7A, the expression of the miRNA novel-m0083-3p in female gonad was significantly lower than that in male gonad, appearing an opposite expression pattern with *tgfb2b*. To further confirm the regulatory relationship, recombinant luciferase reporter plasmids that contained wild-type and mutant-type binding sites (Figure 7B) were co-transfected with novel-m0083-3p mimics into HEK 293T cells, respectively. Compared with NC group, novel-m0083-3p mimics extremely significantly diminished the relative luciferase activity of the cells transfected with wild-type *tgfb2b* 3′-UTR (Figure 7C). However, no negative influence was detected when the cells were transfected with mutant plasmid (Figure 7C). The results proved that *tgfb2b* could be directly targeted and suppressed by novel-m0083-3p.

## 4. Discussion

TGF-β superfamily members participate in a wide range of cellular processes including cell growth and differentiation. They are reported to be crucial to sex development and gonad function [4,5,13,20]. In *C. semilaevis*, *tgfb2b* showed marked female-biased expression in the gonad, which pointed to its functional involvement in ovarian regulation. Like most sequences of the superfamily in other species [8,14], the gene encoded amino acids with a large prodomain region and a C-terminal mature TGF-β domain. It also comprised several conserved cysteine residues, which were helpful to form disulfide bonds, fold stabilized dimer, and then bind to specific receptor [14]. The conserved structure might be a key to the biological activity of this molecule.

In this study, five TGF-β paralogs of *C. semilaevis* were analyzed. They showed various expression patterns in different tissues. Among them, *tgfb2b* was highest expressed in ovary, while *tgfb1a*, *tgfb2a*, *tgfb3a*, and *tgfb3b* were mainly expressed in other tissues like brain and pituitary. *Tgfb1a* and *tgfb3a* were also quantified to be prominently expressed in skin of the fish, but with low expression level in muscle [21]. Their expressions exhibited time-dependent changes after *Vibrio harveyi* infection [21]. In addition, the genes were also revealed to be involved in early response to heat stress in the brain of female and male *C. semilaevis* [22]. This diversity suggests that TGF-β isoforms may exist with varying functions at different body parts [14].

Many TGF-β superfamily genes including *bmp15*, *gdf9*, *activin*, and *tgfb1* are often observed with high abundance in ovary and serve as modulators of ovarian development/function [8,13,23,24]. In zebrafish, mutation of the oocyte-secreted factor *bmp15* could start normal female development but switch sex to fertile male during the juvenile stage, proving that the factor is required for female sexual development and phenotype maintenance [12]. Another example, *tgfb1*, is detected in ovarian follicles at differing developmental periods, and can influence both gonadotropin- and 17α, 20β-dihydroxyprogesterone-triggered oocyte maturation *in vitro* [13]. However, as a member of TGF-β superfamily, the role of *tgfb2b* in fish ovary is not well understood. Coincidently, in the present study, the gene was tested to be ovary-enriched. Its expression throughout ovarian developmental processes from 3 months to 1.5 years after hatching tended to be gradually increased. It was also examined with localization signals in oocytes of different stages, from early small stage I through stage II and mature stage III. In *C. semilaevis,* at the early developmental period, there is nearly no difference between female and male gonads. Sex determination of the fish is prosed at about 50 days after hatching, and gonadal differentiation is initiated at 56–62 days. Nevertheless, for the reason of hysteresis, cellular differentiation often appears in the ovary at 3–4 months, along with the emergence of ovarian cavity. Afterwards, the oocyte goes on to differentiate, exhibiting obvious differentiation at 6–7 months and reaching sexual maturity at 1.5–2 years old [2,25,26]. Considering the fish ovarian developmental performance and the gene expression profile, these findings further implied the importance of *tgfb2b* in female differentiation and development of *C. semilaevis*.

The promoter activity of *tgfb2b* gene was successfully validated via dual-luciferase assay. After searching for transcription factor binding sites in the promoter region, we found that C/EBPα and c-Jun could efficiently repress its promoter activity. The two factors are known to bind with growth- and sex-related genes, adjusting gene expression and undertaking roles in sex regulation [27,28,29]. The binding of C/EBPα to *cyp3a41* promoter can be impacted by sexually dimorphic growth hormone (GH) secretion and attributes to high gene expression level in female [30]. Moreover, C/EBPα/β could regulate ovulation and luteinization-related genes, affecting ovulation via dose- and preovulatory stage-dependent manners [27]. The transcriptional mediator is also involved in terminal differentiation of granulosa cells during luteinization [31]. Likewise, our findings that C/EBPα suppressed the activity of female-biased *tgfb2b* in *C. semilaevis* supported its potential role in female gonad differentiation and development. Additionally, the expression of mice *tgfb2* is mediated by c-Jun transcription factor and involved in the establishment of Sertoli cell polarity [29]. Another JUN family member, JUNB, can bind to and decrease both the mRNA and protein levels of *tgfb2*, which then functions in cell proliferation and ovarian tumor aggressiveness [28]. Consistent with this, the binding of c-Jun to the promoter of *C. semilaevis tgfb2b* suppressed its transcriptional activity. However, the precise molecular mechanisms by which these transcription factors regulate *tgfb2b* function require further elucidation.

After *in vitro* knockdown of *tgfb2b* in CSO cells, the expression levels of several sex-related genes (e.g., *foxl2*, *smad1*, *smad2*, and *esr2b*) were declined. SMAD proteins are intracellular effectors that can be phosphorylated by TGFβ-stimulated receptors and deliver the signal. Female smad1/5 double conditional knockout (dKO) mice develop fertility defects and granulosa cell tumors with significant expression changes in TGF-β family genes, implying the molecule as part of a crucial pathway in ovary [32]. *Foxl2* is also reported to be modified by TGF-β superfamily members [3,33]. It plays a pivotal role in ovarian differentiation by boosting the expression of *cyp19a* (an important female-related gene encoding aromatase that is responsible for estrogen synthesis) and antagonizing male pathway gene expression [34]. Deficiency of *foxl2* leads to the failure of female gonad development, even female-to-male sex reversal, and the mutant phenotype could be rescued by 17β-estradiol (E2) [34,35]. In *C. semilaevis*, *foxl2* and *esr2b* were both downregulated in response to RNAi of *tgfb2b*. The data indicated that TGF-β/SMAD signaling contributed to the fish female development, potentially via regulation of *foxl2*-*cyp19a*-*esr* axis.

Dual-luciferase reporter assay analysis proved that *C. semilaevis tgfb2b* could be bound and negatively regulated by the miRNA novel-m0083-3p. It was consistent with their converse expression profiles in gonads. In other species, *tgfb2* is also reported to be targeted and transcriptionally or post-transcriptionally controlled by miRNAs [36,37,38]. Inhibition of *tgfb2* by miR-301a-5p participates in germline cell differentiation and spermatogenesis [36], while regulation of miR-31 on *tgfb2* and *smad2* affects the developmental potential of oocytes [37]. Similarly, our results suggested the relevance of miRNA in TGF-β pathway-joined sex development of the fish. The detailed role and action mechanism should be forwardly examined.

## 5. Conclusions

In summary, *tgfb2b* exhibited female-dominant expression in gonad of *C. semilaevis*. It was continuously expressed and displayed a progressive increase throughout ovarian developmental periods from 3 months to 1.5 years after hatching. Transcriptional regulation, siRNA knockdown, and miRNA-targeted experiments supported that the gene played a considerable role in ovary differentiation and development. Further investigations on the crosstalk between *tgfb2b* and *foxl2*-*cyp19a*-*esr* will be essential to elucidate the broader sex-determination regulatory network in fish.

## Figures and Tables

**Table 1:** All primers used in the study.

Primer	Sequence (5′-3′)	Information
sex-F	CCTAAATGATGGATGTAGATTCTGTC	sex identification
sex-R	GATCCAGAGAAAATAAACCCAGG	sex identification
*tgfb2b*-CDS-F	ATGTGGCTGCCCCGCCTG	CDS cloning
*tgfb2b*-CDS-R	TCAGCGACACTTGCAGGA	CDS cloning
*tgfb2b*-qF	GCCAAGAAGAACCGCAAGAA	qPCR
*tgfb2b*-qR	TGAGGTCACGCCTGAAGTC	qPCR
*actin*-qF	TTCCAGCCTTCCTTCCTT	qPCR
*actin*-qR	TACCTCCAGACAGCACAG	qPCR
*tgfb2b*-SP6-F	ATTTAGGTGACACTATAGAAACAGAGTCGTCCAGTTCGACGTCA	ISH
*tgfb2b*-T7-R	TAATACGACTCACTATAGGGAGACACGCCTGAAGTCGATGTAGAGCGA	ISH
*tgfb2b*-pF	AGATCTGCGATCTAAGTAAGCTTGGAAAAGTTCCCTCCAGTG	promoter cloning
*tgfb2b*-pR	CAACAGTACCGGAATGCCAAGCTTGCTGTGTGATGTGACTCCA	promoter cloning
*tgfb2b*-C/EBPα-muF	TTTAGAAATCAAGGCAGGGCAGATG	promoter mutation
*tgfb2b*-C/EBPα-muR	CATCTGCCCTGCCTTGATTTCTAAA	promoter mutation
*tgfb2b*-cJUN-muF	CAGGATAATGCTGAGTCATCACACAG	promoter mutation
*tgfb2b*-cJUN-muR	CTGTGTGATGACTCAGCATTATCCTG	promoter mutation
*foxl2*-qF	GAGAGGAAGGGCAACTACTGGA	qPCR
*foxl2*-qR	TGGTTGGAAGTGCGTGGG	qPCR
*smad1*-qF	GTGTCCCACAGGAAAGGTCT	qPCR
*smad1*-qR	TGTAGTGATAGGGGTTGATGC	qPCR
*smad2*-qF	ACATATAGGTCGCGGTGTGAG	qPCR
*smad2*-qR	CCTGATGGTGCACATCCTAGT	qPCR
*esr2b*-qF	GTGGACCATCCTGGGAAACTC	qPCR
*esr2b*-qR	GCTTCAGCTCACGGAATCGA	qPCR
*tgfb2b*-0083-F	ACGCTCGAGACAGGTGAACTTAACAGCCG	plasmid construction
*tgfb2b*-0083-R	ACGGTCGACTCCGAACAGAACATAATCACC	plasmid construction
*tgfb2b*-0083-muF	AAGAGGTATTAAAAGGTTGACACCCAAAAGATCCATGTTTACT	plasmid construction
*tgfb2b*-0083-muR	AGTAAACATGGATCTTTTGGGTGTCAACCTTTTAATACCTCTT	plasmid construction

**Table 2:** RNA oligonucleotides used in the study.

Name	Sense (5′-3′)	Antisense (5′-3′)
*tgfb2b*-siRNA1	GGGACAGAUUCUCAGUAAATT	UUUACUGAGAAUCUGUCCCTT
*tgfb2b*-siRNA2	AAGUGAUGUUGCUCUACAATT	UUGUAGAGCAACAUCACUUTT
*tgfb2b*-siRNA3	AAGGUUACAAAGCCAACUUTT	AAGUUGGCUUUGUAACCUUTT
novel-m0083-3p mimics	UUUUCUGACUAUAAACUGACC	UCAGUUUAUAGUCAGAAAAUU

## Data Availability

The data presented in this study are available in the article.

## Supplementary Materials

The following supporting information can be downloaded at: https://www.mdpi.com/article/10.3390/biom16010105/s1, Table S1: Accession numbers of TGFBs used in the study.
