# Supplementary material for: Identification and Functional Analysis of tgfb2b Gene in Ovarian Development of Chinese Tongue Sole (Cynoglossus semilaevis)
Source: Biomolecules. 2026 Jan 7;16(1):105. doi: 10.3390/biom16010105 (PMC12839324; doi:10.3390/biom16010105)
Supplement: Supplementary file 1 [file biomolecules-16-00105-s001.zip › biomolecules-4049462-supplementary.pdf]

Supplementary Table S1. Accession numbers of TGFBs used in the study

Species	Protein	Accession number
Cynoglossus semilaevis	TGFB1a	XP_008307032.1
	TGFB2a	XP_008322101.1
	TGFB2b	XP_008325099.1
	TGFB3a	XP_008305524.1
	TGFB3b	XP_008311127.1
Homo sapiens	TGFB1	NP_000651.3
	TGFB2	NP_001129071.1
	TGFB3	NP_003230.1
Mus musculus	TGFB1	NP_035707.1
	TGFB2	NP_033393.2
	TGFB3	NP_033394.2
Gallus gallus	TGFB1	NP_001305385.1
	TGFB2	NP_001026216.2
	TGFB3	NP_990785.2
Danio rerio	TGFB1a	NP_878293.1
	TGFB1b	XP_692338.3
	TGFB2a	NP_919366.1
	TGFB2b	XP_021336789.1
	TGFB3a	NP_919367.2
Oryzias latipes	TGFB1a	XP_004075270.1
	TGFB2b	XP_004073197.1
	TGFB3a	XP_004082378.1
	TGFB3b	XP_004083364.1
Oreochromis niloticus	TGFB1a	NP_001298254.1
	TGFB2a	NP_001298243.1
	TGFB2b	XP_003444651.1
	TGFB3a	XP_003453117.1
	TGFB3b	XP_003455646.1
Takifugu rubripes	TGFB1a	NP_001266976.1
	TGFB2a	XP_003965777.1
	TGFB2b	XP_003970254.1
	TGFB3a	XP_003962498.1
	TGFB3b	XP_003971715.1
Ctenopharyngodon idella	TGFB1a	XP_051718112.1
	TGFB1b	XP_051733539.1
	TGFB2a	XP_051720871.1
	TGFB2b	XP_051716562.1
	TGFB3a	XP_051722891.1
Ictalurus punctatus	TGFB1a	XP_017351607.1
	TGFB1b	XP_017349102.1
	TGFB2a	XP_017331706.2
	TGFB2b	XP_017329739.1

	TGFB3a	XP_017331981.1
Anarrhichthys ocellatus	TGFB1a	XP_031719527.1
	TGFB2a	XP_031729706.1
	TGFB2b	XP_031718671.1
	TGFB3a	XP_031702982.1
	TGFB3b	XP_031726318.1
